# Religious affiliation and major depressive episode in older adults: a cross-sectional study in six low- and middle- income countries

**DOI:** 10.1186/s12889-019-6806-1

**Published:** 2019-04-30

**Authors:** Julian A. Fernández-Niño, Ietza Bojorquez, Carolina Becerra-Arias, Claudia I. Astudillo-Garcia

**Affiliations:** 1 Department of Public Health, Universidad del Norte, Colombia. Km. 5 Vía Puerto Colombia, Atlantico Barranquilla, Colombia ZP 081007; 2 Department of Population Studies, El Colegio de la Frontera Norte, Mexico, Km. 18.5 Carretera Escénica Tijuana-Ensenada, San Antonio del Mar, CP 22560 Tijuana, BC Mexico; 3 Research Group on Health, Rehabilitation and Work (SARET), Manuela Beltrán University -- Colombia.Sectional Bucaramanga, Calle 33 #27-12, Bucaramanga, Santander Colombia ZP 680002; 4 Psychiatric Services, Secretaria de Salud, Marina Nacional 60, Tacuba, Miguel Hidalgo, ZP 11410 Ciudad de México, Mexico Mexico

**Keywords:** Religion, Major depressive episode, Depression, Older adults, Minority groups

## Abstract

**Background:**

The relationship of religious affiliation and mental health is complex, and being part of a minority religious group could have negative effects on mental health. In this study, we assessed the association between religious affiliation and major depressive episode (MDE) in older adults (> = 60 years) from China, Ghana, India, Mexico, Russia and South Africa.

**Methods:**

We conducted a secondary analysis of data from the Study on global Ageing and adult health (SAGE), with six nationally-representative community-based samples (*n* = 21,410). Religious affiliation was self-reported by participants, and we defined MDE based on ICD-10 classification. We estimated the association of MDE with religious affiliation versus no religious affiliation, and minority versus majority affiliation.

**Results:**

We observed no association between having a religious affiliation (vs. no affiliation) and the odds of MDE in older adults. In most cases minorities had higher odds of MDE as compared with the majority religion, but the associations were only significant for Muslims in Ghana and for Muslims, Hindus and Other in South Africa.

**Conclusions:**

While the results were significant only for two countries, we observed higher odds of MDE among minorities in most of them. Older adults who are members of religious minorities might be at risk for mental health problems, and there is a need for public health interventions aimed at them.

**Electronic supplementary material:**

The online version of this article (10.1186/s12889-019-6806-1) contains supplementary material, which is available to authorized users.

## Background

A major depressive episode (MDE) is characterized by a combination of depressive symptoms and significant distress or impairment in social life [1]. MDE is the defining component of major depressive disorder, a mental health problem that affects 10–20% of older adults worldwide [2], was the fourth cause of years lived with disability (YLD) for this age group in 2017 [3], and is a growing public health issue in low- and middle-income countries [4]. While MDE and other types of depressive symptoms are not in themselves enough to define a psychiatric disorder such as major depressive disorder, they are part of a range of conditions that impact on well-being. In older adults, MDE increases the risk of suicidal ideation, suicide attempt and suicide [5, 6]. Older adults with depressive symptoms also have a poorer prognosis in other chronic pathologies [7] and increased disability [8].

The etiology of major depressive disorder includes the interactions of diverse biological, psychological and social factors [9], of which religion can be one. Various studies have found inverse associations between religion and depression, with participants reporting a religious affiliation, being involved in church-related activities and with stronger religious beliefs generally having better mental health indicators [10–14]. On the other hand, aging is accompanied by several significant changes such as widowhood and retirement, and social networks tend to decrease over time. Religious affiliation and beliefs could be particularly important to face these social stressors and relevant life changes at this age [15]. In older adults, religion has been associated with better quality of life [16] and lower risk of depression [17–20].

However, the relationship between religion and depression is complex, with differing associations depending on which indicator of religious involvement is measured. As an example, in one study feeling that one received guidance from religion was associated with lower odds of developing depression, but religious attendance was not [11]. Other studies found that attendance was inversely associated with depression [21, 22]. By contrast, positive associations between religion and depressive symptoms have also been reported [13, 23–25], and also U-shaped associations between organizational religious activity and depressive symptoms, and between private religious involvement and depressive symptoms [26]. Still others have failed to find an association between religion-related beliefs and behaviors and depression [21, 27]. It has also been reported that religion-related aspects have a different association with depressive symptoms, depending on the specific religious affiliation (including no affiliation) of individuals [28].

The potential protective effect of religion on mental health might be due to an increase in social networks and social contact through participation in church activities [9], and to the sense of transcendence associated with religious beliefs [14]. On the other hand, a possible explanation for the findings of a positive association between religion and depressive symptoms could be that religious affiliation can also be accompanied by conflict, either with the family or with the larger society, resulting in stress and depressive symptoms [29, 30].

One religion-related aspect that might be associated with mental health is the experience of being a member of a religious minority (defined as a religion with less members than others in a given country) [31] The health of minorities (by national origin, sexual orientation, ethnicity, etc.) can be affected by stigma, discrimination and outright violence [32, 33], and discrimination has well known negative effects on mental health [34, 35], making discrimination a source of health inequity. Religion and ethnicity can also be conflated, minority groups can be marginalized for both reasons, and ethnic conflict can be intertwined with religious conflict [36, 37]. Thus, religious minority members can experience open or covert discrimination and even physical violence, but the study of the health effects of being a minority religion member is relatively recent [33]. To the best of our knowledge, there are no studies of the association of minority religion membership and MDE in older adults, but one survey in this age group in China found that Christians (a religious minority) had higher odds of suicide attempt in the past 12 months, as compared with those without a religious affiliation (Buddhist and Muslim affiliations had no association with suicide attempt) [38]. Similarly, a study of adolescents in Pakistan reported that religious minority members had more depressive symptoms [39] and lower self-esteem [40] than members of the majority religion. On the other hand, a study with data of 10 Catholic-majority countries found that minority religion members that were actively engaged with their religious tradition had better mental health than those who weren’t, but when Catholics represented 90% or more of the population, the opposite was true [33]. Exploring the associations between minority religious affiliation and mental health in a diversity of settings is important to increase our understanding of these relationships.

In this article, we investigate the association between religious affiliation (RA) and MDE in older adults in six lower- and middle-income countries: China, Ghana, India, Mexico, Russia and South Africa. The countries were part of the Study on global AGEing and adult health (SAGE), and together they represent a variety of politic and economic contexts, and are also diverse in terms of the current state and historical evolution of religious faiths. In China, the majority of the population declare having no religious affiliation. There are no major inter-faith conflicts, but Tibetan Buddhists and Muslim groups have clashed with the government. In Ghana, Christians are the religious majority, and there are some tensions between them and the Muslim minority. In India, the relationship between the Hindu majority and Muslims has been difficult and even violent at some points, and Muslims have been victims of discrimination by the government. In Mexico, the relationship between members of the Catholic majority and religious minorities (mainly Evangelical Christian) is mostly peaceful, with some violent conflicts over land ownership between Catholics and Evangelical Christian groups in rural areas. In Russia, since the end of the Soviet Union, Orthodox Christianism returned to a prominent position as the main religion in the country. Finally, in South Africa Christianism is the majority religion, with few interfaith conflicts in the recent past.

The objectives of this analysis were: 1) To explore the association between RA and MDE, with the hypothesis that having a RA, versus having no RA, would be protective for mental health; 2) To explore the association between minority RA and MDE, with the hypothesis that having a minority RA, versus having a majority RA, would be a risk factor for mental health problems.

## Methods

### Sample and data collection

Data came from the first wave of SAGE, conducted in 2007–2010, with a sample of 34,124 participants 50 years old or older, and 8340 participants between 18 and 49 years old. The study included six low- and middle-income countries: China, Ghana, India, Mexico, the Russian Federation and South Africa. In this article, we analyze data from older adult participants (aged 60 or older [41]) (*n* = 21,410). The SAGE sampling design included various sampling stages, and was stratified, designed to be representative at the national level for each country included. The ultimate sampling unit were households. All household members aged 50 years or older, and one randomly selected member 18–49 years of age, responded the study questionnaire. The cognitive status of older adults was evaluated by interviewers before applying the questionnaire. When a cognitive impairment was identified, a proxy respondent was utilized and depressive symptoms were not fully evaluated (the proxy respondent only answered if the older adult had been sad). Cognitive limitations were identified in 1.8% of the sample. For this analysis, only questionnaires responded by the older adult him/herself were employed. All data collection procedures were standardized, and staff was previously trained to conduct procedures. Wave 0 of SAGE corresponded to the World Health Survey of 2002–2004 [42]. The response rates for the first wave after that, calculated as participation in the first wave with respect to potential participants identified in wave 0, were 93% for China; 83% for Russia; 68% for India; 81% for Ghana, 53% for Mexico; and 74% for South Africa. The main reason for non-response in SAGE first wave was loss to follow-up from wave 0, mainly due to lack of time during fieldwork to locate participants in the World Health Survey [43], and analysis of the association between non-response and risk factors for depression has shown that the non-response was non-differential [44]. More information on sampling and procedures can be consulted in a previous publication [42].

Four questionnaires were applied as part of the SAGE study: 1) a household questionnaire for information about household chores distribution, house conditions and household socioeconomic position; 2) an individual questionnaire inquiring about health and health determinants, disability, work history, chronic conditions, risk factors, health services response, caregivers and subjective wellbeing; 3) a proxy-respondent questionnaire for health, functional status, chronic conditions and health services utilization; and 4) a module of verbal autopsy to determine probable cause of death of members of the household during the previous 24 months. The questionnaires were developed for SAGE, and are available in the World Health Organization’s web page at http://apps.who.int/healthinfo/systems/surveydata/index.php/catalog/5.

### Outcome

The questionnaire of SAGE included an abbreviated version of the World Mental Health Survey version of the Composite International Diagnostic Interview (WMH-CIDI), which asks about history of depressive episodes in the last year, as well as depressive symptoms in the last two weeks. We followed an algorithm developed and previously used by the SAGE team [45] that combines scores on the WMH-CIDI questions to obtain an indicator of MDE according to DSM-IV criteria. The concordance of WHM-CIDI with clinical assessments has been evaluated as moderate to good [46]. For the algorithm, we classified symptoms in two sets. Set A included six items measuring three domains: depressive affect, anhedonia (loss of interest or pleasure in activities, including decreased libido), and tiredness or lack of energy. Set B included 12 items measuring seven domains (1–2 items per domain): hopelessness, low self-esteem or unworthiness; anxiety or psychomotor agitation; suicidal attempt or ideation; slow thinking or lack of concentration; slow movement; sleep alterations; and appetite loss. For each domain in each set, a dichotomous variable was computed as 1 when the participant responded affirmatively to at least one of the items in the domain, and 0 otherwise. Then, a score was obtained for each set. A participant was considered to have had a MDE if he/she scored > = 2 points in set A and > =4 points in set B.

### Independent variable

The main independent variable was religious affiliation, assessed in the SAGE questionnaire by the question “Do you belong to a religious denomination?” The response categories were: unaffiliated, Buddhist, Traditional Chinese Religion, Christian, Hindu, Muslim, Jewish, Indigenous, Sikh, Other. For this analysis, we kept the categories unaffiliated, Buddhist, Christian, Hindu and Muslim, and grouped the rest as “Other” (a category that comprised less than 0.5% in each country). While there is no general agreed-upon definition of what a minority is, a rough definition might be that minorities are social groups that are numerically inferior to others in a given country [31]. For this analysis, we defined minority religious affiliation by country, by comparison to the affiliation declared by the majority of participants.

### Potential confounders

The models were adjusted by variables related to both religious affiliation and MDE, including sociodemographic, household, health conditions and social networks variables.

The sociodemographic variables were gender, age (in categories 60–69, 70–79 and 80+ yrs.), living with a partner (either by marriage or de facto), educational attainment (none, incomplete or complete elementary school, incomplete or complete secondary school, and college or higher), and having health insurance coverage.

We also considered socioeconomic position, defined as the relative position in household wealth. We used the methodology previously described by Fernández-Niño et al. [47], computing an index from 20 items asking for ownership (yes or no) of various goods at the household level. After a factor analysis with a tetrachoric correlation matrix of the 20 items, we obtained the prediction of the first factor (explaining 46% of the joint variance of the items) and categorized it by country-specific tertiles, where the first tertile corresponded to the lower socioeconomic position and the third to the higher socioeconomic position.

Health-related variables included multimorbidity and physical disability. As in a previous work [47], we computed a variable with categories from zero to three or more diagnosis (the standard definition of multimorbidity) [48], from the self-report of a medical diagnosis of arthritis, cerebrovascular event, angina, diabetes, chronic obstructive pulmonary disease, asthma, hypertension, or cataracts. Physical disability was a yes/no indicator of being severely or extremely limited in at least one of 18 basic activities over the previous 30 days, an approach based on the World Health Organization’s Disability Assessment Schedule (WHODAS 2.0) [45, 49].

Finally, we included three variables, each one representing a different domain of social networks: 1) not living alone; 2) participation in non-religious social activities (being a member of at least one social organization other than a church); and 3) having someone to trust. All of the above variables were dichotomic (yes = 1 and no =0).

### Statistical analysis

We conducted exploratory analyses with central tendency (mean or median) and dispersion measures (standard deviation and interquartile range) for quantitative variables, and proportions and confidence intervals at the 95% level (CI 95%) for categorical variables. We estimated the standardized prevalence rate of MDE with the direct method, using as population of reference the world population provided by the World Health Organization [50]. We conducted bivariate analysis of the associations between the dependent variable and each independent variable with chi-square tests and Mann-Whitney U tests.

To estimate the association of religious affiliation and MDE we employed logistic regression models, and report the results as odds ratios (OR) and their respective CI 95% and *p*-values. We estimated two models: one comparing the unaffiliated vs each religious affiliation, and another comparing the majority religion for each country vs the other religious affiliations and the unaffiliated.

All analyses considered the complex sampling design, and were done with Stata 14 (Stata Corporation, College Station, TX, USA).

### Ethical considerations

All participants in SAGE signed and informed consent. Approval for the SAGE study was obtained from institutional review boards in each participant country, and endorsed by the World Health Organization’s committee. This article presents the results of a secondary analysis of data from that study. The information collected by wave 1 of the SAGE study is public. Authorization from SAGE was obtained for the present analysis via the internet.

## Results

Women comprised 53.8% of the study sample. The mean age of the study sample was 69.9 (SD = 7.6) years, with most participants in the 60–69 years range. As for educational attainment, 33.9% of participants reported no formal education, 33.7% had primary education or less, 25.7% had secondary education, and 6.7% had some college education or more. In Table 1, we present the general characteristics of the sample, by gender and presence of a MDE in the past 12 months. Among both men and women, those with a MDE had lower educational attainment, were less likely to have health insurance, were in the lower tercile of household wealth, were more likely to live in rural zones and to have a physical disability. They were also less likely to participate in non-religious social activities, and to have someone to trust. Finally, those with a MDE in the past 12 months were more likely to have a religious affiliation (as opposed to no affiliation).

**Table 1:** Characteristics of older adults in SAGE participant countries, 2007–2010, by major depressive episode (MDE) during the past 12 months

Variable	Men		*p*-value^a^	Women		*p*-value^a^
	MDE (*n* = 491)	No MDE (*n* = 8584)		MDE (*n* = 790)	No MDE (*n* = 9816)	
Sociodemographic						
Age						
60–69	55.3	56.8	0.88	49.5	52.7	0.46
70–79	35.3	34.7		37.6	36.6	
≥ 80	9.4	8.5		12.9	10.7	
Living with a partner	85.3	86.3	0.77	47.3	57.0	< 0.01
Educational attainment						
None	48.4	22.8	< 0.01	70.9	48.6	< 0.01
Elementary	31.7	40.1		17.2	27.8	
Secondary	17.3	29.7		10.5	20.4	
College or higher	2.7	7.3		1.4	3.4	
Having health insurance coverage	19.6	66.7	< 0.01	21.8	65.6	< 0.01
Household wealth (terciles)						
1 (lower)	46.6	31.9	< 0.01	44.0	34.8	0.01
2	36.0	35.0		31.4	35.2	
3 (higher)	17.4	33.1		24.6	30.0	
Zone						
Urban	30.3	42.7	< 0.01	33.1	44.8	< 0.01
Rural	69.7	57.3		66.9	55.2	
Health-related						
Multimorbidity						
None	37.6	48.3	0.04	40.5	41.7	0.16
One	32.2	29.4		27.0	31.0	
Two or more	30.2	22.2		32.5	27.3	
Physical disability	77.6	27.1	< 0.01	85.2	38.5	< 0.01
Social networks						
Not living alone	94.1	92.1	0.35	89.1	82.8	< 0.01
Participation in non-religious social activities	17.6	26.3	< 0.01	8.4	16.6	< 0.01
Having someone to trust	86.0	93.3	< 0.01	74.7	88.1	< 0.01
Religious affiliation						
Affiliation						
None	13.3	63.8	< 0.01	14.0	54.6	< 0.01
Buddhist	4.6	2.1		1.9	4.7	
Christian	4.0	6.0		8.8	12.6	
Hindu	59.2	23.9		64.6	23.8	
Muslim	17.8	3.7		9.0	3.6	
Other	1.1	0.6		1.9	0.8	

^a^*p*-value for the difference between the groups with and without major depressive episode (MDE)

In the sample as a whole, the majority of participants (37.7%) reported having no religious affiliation. The next more frequent affiliation was Christian (27.1%) followed by Hindu (16.5%). However, these unadjusted percentages have no straightforward interpretation, as the sample from China (where most participants were unaffiliated) was larger than the others and therefore descriptive statistics for the whole sample are mainly reflective of that sample. Table 2 shows the frequencies of each affiliation by country.

**Table 2:** Religious affiliation of older adults in SAGE participant countries, 2007–2010

Religious affiliation	China (*n* = 7208)	Ghana (*n* = 2608)	India (*n* = 3621)	México (*n* = 1781)	Rusia (*n* = 2439)	Sudáfrica (*n* = 1847)
	n (%)					
None	6579 (92.6)	140 (5.3)	7 (0.2)	53 (2.9)	423 (13.5)	114 (6.9)
Buddhist	418 (4.9)	3 (0.2)	39 (1.5)	0	0	8 (0.6)
Christian	155 (2.0)	1750 (67.2)	23 (0.4)	1615 (91.0)	1732 (78.6)	1544 (83.8)
Hindu	0	1 (0.1)	3082 (85.6)	39 (1.8)	0	61 (1.6)
Muslim	24 (0.2)	409 (16.5)	416 (11.2)	14 (1.1)	259 (6.7)	56 (2.3)
Other	32 (0.3)	305 (10.7)	54 (1.1)	60 (3.3)	25 (1.2)	64 (4.8)

The 12-month prevalence of MDE was 6.5% (CI 95% 6.2–6.9) for the whole sample, 7. 5% (CI 95% 7.0–8.0) among women and 5.4% (CI 95% 4.9–5.9) among men. The prevalence of MDE by country and religious affiliation appears in Table 3. In China, Muslims had a significantly higher prevalence as compared with the non-affiliated and Buddhists. In Ghana, Muslims had a significantly higher prevalence as compared with Christians. In other countries there were no statistically significant differences in the prevalences.

**Table 3:** Twelve-month prevalence of major depressive episode by country and religious affiliation among. older adults in SAGE participant countries, 2007–2010

Religious affiliation	China	Ghana	India	México	Rusia	Sudáfrica
	% (CI 95%) ^a^					
None	1. 6 (1.2–1.9)	11.8 (4.4–19.1)	NE^a,b^	6.0 (0.8–12.9)	5.2 (2.6–8.4)	1.92 (0.4–4.2)
Buddhist	1.2 (0.1–2.4)	NE^b^	35.3 (5.9–64.7)	NE^b^	NE^b^	NE^b^
Christian	2.2 (1.1–5.5)	8.5 (7.1–9.9)	11.6 (9.2–32.5)	6.3 (4.4–8.3)	5.6 (3.5–7.5)	2.00 (0.4–3.6)
Hindu	NE^b^	NE^b^	15.8 (13.8–17.8)	9.7 (0.7–20.0)	NE^b^	7.1 (1.0–15.1)
Muslim	2.6 (2.5–7.7)	14.1 (9.9–18.4)	22.8 (16.1–29.5)	10.3 (4.6–25.2)	7.5 (2.3–12.6)	8.9 (5.3–23.0)
Other	NE^b^	8.2 (4.8–11.7)	24.5 (7.6–41.4)	15.9 (0.6–32.4)	6.7 (2.5–15.8)	13.3 (1. 9–28. 5)

^a^Percentages and confidence intervals represent the prevalence of mayor depressive episode for each combination of country and religion. ^b^ NE: Non-estimable

The crude logistic regression model for the whole sample (Table 4, unadjusted) showed that, contrary to our expectation, religious affiliation was associated with higher odds of MDE for all categories of affiliation, as compared with unaffiliated participants. However, after adjusting by covariables, the effect was no longer statistically significant (Table 4, adjusted).

**Table 4:** Models for major depressive episode among older adults in SAGE participant countries, 2007–2010

Religious affiliation	Unadjusted model			Adjusted model^a^		
	OR	CI 95%	*p*-value	OR	CI 95%	*p*-value
None	Reference					
Buddhist	3.8	1.3–11.0	0,013	1.3	0.6–2.7	0.493
Christian	3.2	2.1–4.7	< 0,0001	0.9	0.4–1.9	0.823
Muslim	11.3	8.5–15.0	< 0,0001	0.6	0.3–1.4	0.290
Hindu	14.9	9.7–22.8	< 0,0001	0.8	0.3–1.9	0.724
Other	9.5	4.7–19.1	< 0,0001	1.1	0.4–3.2	0.874

^a^Adjusted by all variables that appear in Table 1

In Tables 5 and 6 we show the results of logistic regression models stratified by country. With the unaffiliated as the reference category (Table 5), we observed significantly higher odds of MDE only for Muslims in Ghana (OR 2.6; CI 95%: 1.1–6.4). When using the majority affiliation as the reference category (Table 6), Muslims in Ghana again still had higher odds of MDE (OR 2.7; CI 95%: 1.7–4.1). In this analysis, minorities in South Africa also had higher odds of MDE (Hindus OR 14.8, CI 95%: 2.7–82.4; Muslims OR 53.9, CI 95%: 5.5–525.2; other OR 18.7, CI 95%: 4.7–74.9).

**Table 5:** Adjusted odds ratios (OR) ^a^ for major depressive episode among older adults in SAGE participant countries, 2007–2010. Unaffiliated as reference category

Religious affiliation	China	Ghana	India	Mexico	Russia	South Africa
	OR (CI 95%)					
None	Reference					
Buddhist	0.7 (0.2–2.1)	NE	1.6 (0.3–8.0)	NE	NE	NE
Christian	1.6 (0.3–8.1)	1.4 (0.6–3.3)	0.5 (0.0–5.8)	0.7 (0.2–2.5)	1.1 (0.5–2.3)	0.7 (0.1–3.2)
Hindu	NE	NE	0,6 (0.2–1.8)	1.2 (0.2–6.7)	NE	2.1 (0.4–12.2)
Muslim	2.4 (0.3–16.8)	**2.6 (1.1–6.4)**	0.8 (0.2–2.5)	1.1 (0.1–9.2)	1.1 (0.4–3.1)	2.9 (0.2–37.7)
Other	NE	1.5 (0.6–3.7)	NE	1.6 (0.3–8.5)	1.8 (0.4–8.2)	7.1 (0.9–52.2)

^a^Adjusted by all variables that appear in Table 1. Odds ratios in each column are within-country. NE: Non-estimable

The significance of the boldface data is indicated by the CI 95% not including the null value of 1

**Table 6:** Adjusted odds ratios (OR) ^a^ for major depressive episode among older adults in SAGE participant countries, 2007–2010. Majority religion as reference category

Religious Affiliation	China	Ghana	India	Mexico	Russia	South Africa
	OR (CI 95%)					
None	Reference	1.1 (0.5–2.4)	NE	1.6 (0.3–8.2)	0.9 (0.4–2.0)	1.1 (0.2–7.3)
Buddhist	0.7 (0.2–2.2)	NE	2.7 (0.8–8.8)	NE	NE	NE
Christian	1.9 (0.4–8.7)	Reference	0.8 (0.1–7.2)	Reference	Reference	Reference
Hindu	NE	NE	Reference	1.6 (0.6–4.5)	NE	**14.8 (2.7–82.4)**
Muslim	2.1 (0.3–14.7)	**2.7 (1.7–4.1)**	1.3 (0.8–2.0)	1.5 (0.3–8.5)	1.4 (0.6–3.2)	**53.9 (5.5–525.2)**
Other	NE	1.1 (0.6–1.9)	1.5 (0.5–4.8)	2.7 (0.7–11.1)	1.7 0.4–7.4)	**18.7 (4.7–74.9)**

^a^Adjusted by all variables that appear in Table 1. Odds ratios in each column are within-country. NE: Non-estimable

The significance of the boldface data is indicated by the CI 95% not including the null value of 1

## Discussion

We found no evidence of the expected inverse association between religion and MDE. This is in contrast with previous studies that found associations between personal and organized religious practices and religiosity, and lower risk of depression symptoms [17–20, 23]. However, as others have pointed out [13, 26], the association can be different depending of what aspect of religion is measured (religiosity vs spirituality, personal vs organized practices, etc.), and as our study measured only one aspect (affiliation) it is difficult to establish adequate comparisons.

In all six countries included, the prevalence of MDE was either higher or similar among those who reported a religious affiliation, as compared to the unaffiliated. The association between reporting a religious affiliation and higher odds of MDE, however, was explained away by sociodemographic, household and health related variables that were in turn associated with religious affiliation. Additional file 1: Table S1 presents the distribution of other covariates by religion, showing that the unaffiliated were more likely to be male, in the upper quartiles of household wealth, more educated, living with a partner and having someone to trust. All of these characteristics are inversely associated with depression, and so one way of interpreting this result is that the apparent religion-MDE relationship was the result of confounding by other factors.

However, the association of certain characteristics with religion is socially determined. The fact that, in the combined sample for the six countries, Muslims tended to be poorer and having lower educational attainment is the result of sociohistorical conditions, so the sociodemographic and other variables could be interpreted as part of a “cluster of disadvantages” [51] experienced by minority religions. From this point of view, the loss of significance of the effect of religious affiliation after adjustment by covariates doesn’t mean that the “true” relationship is not significant, only that it occurs as part of a complex network of social relationships.

Even after adjusting by other variables, an association remained between minority religion and MDE for Muslims in Ghana, and for Hindus, Muslims and “Other” in South Africa. Religious minorities can experience multiple stressors that offset the positive aspects of religion, but the effect of being a member of a minority religion might also vary depending on the context. The expectation would be that in countries with more interfaith conflict or discrimination on the basis of religion, the negative living experiences associated with minority religion should increase. The importance of context was evidenced by a previous study reporting that active members of religious minorities had worst health and wellbeing indicators when living in countries where the percentage of members of the majority religion was higher [33]. However, according to a recent survey, residents of Ghana and South Africa are not especially likely to cite religious conflict as a very big problem, and while the percentage saying that religion is important to them is high, it is not particularly so when compared with other African countries [52].

In order to understand why the expected association was only significant in Ghana and South Africa, a more in-depth exploration of the contextual differences that affect the experience of being a member of a minority would be required, but we can advance some suggestions. First, our sample was composed of older adults, so the change in religion-related social aspects during their lifetimes could explain the differences more than the current conditions. Second, the small sample size in some groups might have limited the statistical power to uncover associations, although the fact that most of them were in the direction of higher odds of MDE among minorities seems to confirm our hypothesis regarding the association of minority religion and MDE. Third, it is possible that for some countries the relevant majority and minority comparison groups were not assessed. This probably was the case in Mexico and Russia, where a more significant contrast might have been between types of Christian faith that were mixed in the “Christian” category of SAGE (Protestants/Evangelicals vs. Catholics in Mexico, and Orthodox vs. other Christians in Russia). Likewise, the “Other” category in South Africa might have been a very different mixture than the same category in other countries. A more precise investigation of the subject should consider all of these aspects.

A limitation of our study was the cross-sectional design, as the association of religious affiliation and mental health could theoretically go in both directions. Another limitation was that religious affiliation is only one aspect of religion, and doesn’t necessarily indicate spirituality, the importance attributed to religious beliefs, and other elements that the literature has reported as related to depression. Using a variety of measures of religion-related aspects might have changed our results, especially if those aspects are associated with being part of a religious minority. However, with the current data it is difficult to hypothesize in which direction the results would have changed. Also, the percentage of the population affiliated with some denominations is very low for some countries, resulting in zero or a small number of respondents for some combinations of category-country, so that the analysis of the association of specific denominations and MDE might be non-significant due to lack of power. Finally, MDE is an extreme of the continuum between emotional well-being and disorder, so that our results apply to what could be considered the graver cases of depression.

## Conclusion

To conclude, our results suggest an association between minority religion membership and MDE. This finding is important for public health, as it points to a social source of health inequity that could be addressed through public policies protecting minorities. To our knowledge, this is the first study to explore this issue in a sample of older adults from diverse sociocultural contexts. Older adults who are members of religious minorities might be at risk for mental health problems, and there is a need for public health interventions aimed at them.

## Additional file

Additional file 1: **Table S1.** Characteristics of participants by religious affiliation, SAGE round one. **Table S2.** Characteristics of participants by religious affiliation, SAGE round one. (DOCX 20 kb)

## Abbreviations

MDE: Major depressive episode
RA: Religious affiliation
SAGE: Study on global Ageing and adult health
WHODAS: World Health Organization’s Disability Assessment Schedule
