# Supplementary material for: Religious affiliation and major depressive episode in older adults: a cross-sectional study in six low- and middle- income countries
Source: BMC Public Health. 2019 Apr 30;19:460. doi: 10.1186/s12889-019-6806-1 (PMC6492427; doi:10.1186/s12889-019-6806-1)
Supplement: Supplementary file 1 — Table S1. Characteristics of participants by religious affiliation, SAGE round one. Table S2. Characteristics of participants by religious affiliation, SAGE round one. (DOCX 20 kb) [file 12889_2019_6806_MOESM1_ESM.docx]

**Additional file 1: Table S1. Characteristics of participants by religious affiliation, SAGE round one.**

| **Variable** | **None**  **%( n=7314)** | **Buddhist**  **%(n=468)** | **Christian**  **%(n=6814)** | **Hindu**  **%(n=3182)** | **Muslim**  **%(n=1177)** | **Other**  **%(n=540)** | **p-value^1^** |
| --- | --- | --- | --- | --- | --- | --- | --- |
| **Sociodemographic** |  | | | | | | |
| Sex |  | | | | | | |
| Male | 53.4 | 32.3 | 31.6 | 48.2 | 52.1 | 41.0 | <0.01 |
| Female | 46.6 | 67.7 | 68.4 | 51.8 | 47.9 | 59.0 |  |
| Age |  | | | | | | |
| 60-69 | 56.7 | 51.3 | 47.1 | 53.2 | 59.1 | 47.7 | <0.01 |
| 70-79 | 34.8 | 39.6 | 37.8 | 36.2 | 31.1 | 42.8 |  |
| ≥80 | 8.5 | 9.2 | 15.2 | 10.6 | 9.8 | 9.5 |  |
| Living with a partner | 79.3 | 63.4 | 47.9 | 63.4 | 67.5 | 60.1 | <0.01 |
| Educational attaintment |  |  |  |  |  |  |  |
| None | 31.0 | 48.1 | 11.1 | 55.2 | 58.6 | 48.2 | <0.01 |
| Elementary | 39.8 | 34.6 | 19.5 | 25.5 | 23.5 | 24.9 |  |
| Secondary | 23.8 | 15.7 | 59.8 | 15.6 | 14.5 | 20.4 |  |
| College or higher | 5.5 | 1.5 | 9.7 | 3.8 | 3.3 | 6.6 |  |
| Health insurance coverage | 90.2 | 77.8 | 86.5 | 3.2 | 17.2 | 45.6 | <0.01 |

**^1^** p-value for the difference between affiliations

(Continues in next page…)

(… Continued from last page)

**Additional file 1: Table S2. Characteristics of participants by religious affiliation, SAGE round one.**

| **Variable** | **None**  **%( n=7314)** | **Buddhist**  **%(n=468)** | **Christian**  **%(n=6814)** | **Hindu**  **%(n=3182)** | **Muslim**  **%(n=1177)** | **Other**  **%(n=540)** | **p-value^1^** |
| --- | --- | --- | --- | --- | --- | --- | --- |
| Household wealth (terciles) |  | | | | | | |
| 1 (lower) | 32.1 | 35.7 | 34.5 | 35.6 | 47.5 | 38.2 | <0.01 |
| 2 | 36.5 | 37.0 | 35.7 | 31.3 | 36.7 | 25.7 |  |
| 3 (higher) | 31.4 | 27.2 | 29.9 | 33.1 | 15.9 | 36.0 |  |
| Zone |  | | | | | | |
| Urban | 45.1 | 49.7 | 66.2 | 32.0 | 30.5 | 29.0 | <0.01 |
| Rural | 54.9 | 50.3 | 33.8 | 68.0 | 69.5 | 71.0 |  |
| **Health-related** |  | | | | | | |
| Multimorbility |  | | | | | | |
| None | 43.1 | 39.1 | 26.7 | 54.4 | 47.6 | 46.0 | <0.01 |
| One | 32.0 | 32.8 | 26.2 | 26.9 | 31.8 | 31.3 |  |
| Two or more | 25.0 | 28.1 | 47.1 | 18.7 | 20.6 | 22.7 |  |
| Disability | 20.2 | 26.0 | 43.1 | 63.0 | 67.4 | 51.2 | <0.01 |
| **Social networks** |  | | | | | | |
| Not Living alone | 87.2 | 82.5 | 67.5 | 95.5 | 93.3 | 91.8 | <0.01 |
| Participation in non-religious activities | 18.6 | 25.3 | 29.2 | 22.3 | 18.5 | 20.9 | <0.01 |
| Having someone to trust | 97.6 | 95.3 | 81.8 | 77.2 | 80.5 | 89.5 | <0.01 |

**^1^** p-value for the difference between affiliations
